# The impossible confounder: Quantifying the limits of alternative explanations for COVID-19 vaccine effectiveness

**DOI:** 10.1371/journal.pone.0336063

**Published:** 2025-12-04

**Authors:** Tommaso Costa

**Affiliations:** 1 GCS-fMRI, Koelliker Hospital and Department of Psychology, University of Turin, Turin, Italy; 2 FOCUS Laboratory, Department of Psychology, University of Turin, Turin, Italy; 3 Neuroscience Institute of Turin (NIT), Turin, Italy; Drexel University School of Public Health, UNITED STATES OF AMERICA

## Abstract

**Background:**

Observational studies have consistently reported large reductions in COVID-19 risk among vaccinated individuals. However, critics have raised concerns that unmeasured confounding may entirely explain these associations.

**Methods:**

We combined the classical Cornfield inequality with a Monte Carlo sensitivity analysis to evaluate whether unmeasured confounding alone could plausibly account for the observed effectiveness of COVID-19 vaccines. The Cornfield inequality provides a lower bound on the strength of confounding required to explain a given association. The Monte Carlo analysis simulates uncertainty over possible confounder–exposure and confounder–outcome relationships by drawing from weakly informative prior distributions, allowing us to estimate the frequency with which such confounding would be sufficient.

**Results:**

For an observed risk ratio of 0.08—consistent with early estimates for the Pfizer-BioNTech vaccine—the confounder would need to be both highly imbalanced (e.g., 10 times more prevalent among vaccinated individuals) and strongly protective (e.g., reducing disease risk by 99%). Simulation results showed that, under the specified assumptions, fewer than 2% of draws satisfied this condition. Even in the more moderate case of a risk ratio of 0.25 (e.g., AstraZeneca), the proportion remained below 6%.

**Conclusions:**

Our findings suggest that while residual confounding may attenuate effect estimates, it is statistically and epidemiologically implausible that unmeasured confounding alone could fully account for the magnitude of observed vaccine effectiveness. This framework combines the falsificatory logic of Cornfield bounds with the flexibility of simulation-based sensitivity analysis, providing a transparent tool for evaluating confounding-based explanations in observational research.

## 1. Introduction

The COVID-19 pandemic has catalyzed an unprecedented surge in observational research, primarily focused on assessing the practical efficacy of vaccines. While randomized controlled trials (RCTs) have yielded compelling initial evidence of vaccine efficacy, a significant portion of post-marketing data originates from large-scale cohort studies, case-control designs, and retrospective analyses, all of which are susceptible to potential confounding factors. Notably, skeptics of vaccine effectiveness findings have posited that observed disparities in infection or hospitalization rates between vaccinated and unvaccinated individuals may be attributable, at least in part, to unmeasured or uncontrolled confounders rather than a genuine causal impact of the vaccine itself [1,2].

This concern has been exacerbated by the well-documented limitations of observational studies in the field of infectious disease epidemiology. Individuals who opt to receive vaccinations may systematically exhibit distinct characteristics compared to those who do not, including varying health status, risk-taking behaviors, access to healthcare services, and adherence to public health directives. Such disparities could potentially result in an overestimation of vaccine efficacy if the vaccinated cohort also demonstrates a reduced likelihood of virus exposure or heightened engagement in protective measures. Conversely, in certain settings, exposure risk may have been elevated among vaccinated individuals, particularly among healthcare workers and other early recipients of the vaccine, potentially skewing estimates in the opposite direction [3].

These concerns are scientifically valid and warrant rigorous examination. However, it is equally crucial to consider the quantitative magnitude required for an unmeasured confounder to fully account for the observed correlation between vaccination and a decreased risk of COVID-19 outcomes. This is not merely a philosophical inquiry; it is a quantitative one that can be precisely formulated and addressed employing conventional epidemiological methodologies. One such tool is the Cornfield inequality, initially introduced in the 1950s to evaluate the plausibility of confounding as an alternative explanation for the pronounced association observed between cigarette smoking and lung cancer [4].

The Cornfield inequality offers a straightforward yet potent criterion: for a single binary confounder to account for a given risk ratio, the product of its association with the exposure and with the outcome must be at least as substantial as the observed risk ratio. In other words, unless the confounder is both significantly more prevalent among the exposed (in this instance, the vaccinated) and strongly correlated with the outcome (infection or hospitalization), it cannot fully account for the observed effect.

In this study, we apply the Cornfield inequality to recent observational estimates of COVID-19 vaccine effectiveness, utilizing published risk ratios from real-world studies as benchmarks. We extend the analysis employing a sensitivity approach, which enables us to surpass deterministic bounds and assess the probability that an unmeasured confounder could plausibly account for the observed data, given plausible prior assumptions. Our objective is not to assert the absolute veracity of vaccine efficacy—an ontological claim—but rather to evaluate the robustness of the empirical evidence to the specific challenge of unmeasured confounding.

We posit that integrating classical epidemiological principles with uncertainty quantification provides a lucid and transparent framework for addressing methodological skepticism. Instead of disregarding concerns regarding confounding, we quantify the severity of those confounders required to manifest a significant impact. By doing so, we offer a principled response to the assertion that “correlation does not imply causation,” demonstrating that not all correlations are equally amenable to explanation.

Although the selected VE estimates refer to early variants of SARS-CoV-2, the analysis serves to illustrate the plausibility of confounding-based explanations across a range of effect sizes and can be extended to more recent data.

Notably, in some early vaccination settings, confounding may have been negative—for example, due to increased exposure among healthcare workers. This would bias VE estimates downward and make our analysis conservative in its conclusions.

## 2. Materials and methods

To evaluate the plausibility that an unmeasured confounder could account for the observed efficacy of COVID-19 vaccines, we integrated two complementary methodologies: a conventional epidemiological inequality and a probabilistic framework. The former relies on the Cornfield condition, which establishes a deterministic limit on the strength of a confounder required to negate an observed association. The latter employs a Monte Carlo sensitivity analysis, which enables us to compute the probability that a confounder could fulfill that condition under a spectrum of plausible prior assumptions.

### 2.1. Observational estimates of vaccine effectiveness

We selected representative risk ratios (RRs) from real-world studies and meta-analyses of COVID-19 vaccine effectiveness, primarily focusing on symptomatic infection as the primary endpoint. For the Pfizer-BioNTech and Moderna vaccines, risk ratios as low as 0.08–0.10 have been reported across various settings [5,6]. For vector-based vaccines such as AstraZeneca and Johnson & Johnson, typical estimates range from 0.25 to 0.40 [7,8]. These estimates represent the ratio of infection rates in vaccinated versus unvaccinated individuals, adjusted for known covariates. Our analysis assumes these values as observed benchmarks and investigates whether unmeasured confounding alone could plausibly generate them.

We focused on symptomatic infection as it is the most commonly reported outcome in early observational studies, providing a stringent test for confounding-based explanations.

### 2.2. Cornfield inequality: Minimum conditions for confounding

The Cornfield inequality expresses a necessary condition for an unmeasured binary confounder to fully account for an observed risk ratio $RR_{\text{obs}}$. Let $RR_{CE}$ denote the risk ratio between the confounder and the exposure (e.g., how much more common the confounder is among vaccinated individuals), and let $RR_{CD}$ denote the association between the confounder and the disease outcome. Then, the product of these two quantities must satisfy the inequality:


$$RR_{CE}\cdot RR_{CD}\geq RR_{\text{obs}}.$$

Rewriting this as:


$$RR_{CD}\geq \frac{RR_{\text{obs}}}{RR_{CE}},$$

we can evaluate how strong $RR_{CD}$ must be for a confounder to explain the observed data, given various plausible values of $RR_{CE}$. In practice, we computed the required minimum value of $RR_{CD}$ for a range of $RR_{CE}$ values from 1.1 to 10, corresponding to confounders that are slightly to strongly more prevalent among the vaccinated. This analysis provides a simple yet stringent test of the plausibility of confounding explanations.

### 2.3. Monte Carlo sensitivity analysis with prior assumptions

While the Cornfield bound provides a deterministic threshold, it does not quantify uncertainty or account for distributions of possible confounder strengths. To address this, we implemented a Monte Carlo sensitivity analysis in which we simulate uncertainty by drawing from prior distributions over the two latent parameters $RR_{CE}$ and $RR_{CD}$. This approach allows us to estimate the proportion of scenarios in which unmeasured confounding could fully account for the observed effect. While not a full Bayesian model involving a likelihood or posterior updating, this approach enables probabilistic reasoning over the space of possible confounding configurations.

Specifically, we assumed log-uniform priors for both parameters:


$$\text{log}({\text{RR}}_{CE})\sim \text{Uniform}[\text{log}(\text{1}),\text{log}(\text{1}\text{0})]$$

$$\text{log}({\text{RR}}_{CD})\sim \text{Uniform}[\text{log}(\text{0}.\text{0}\text{1}),\text{log}(\text{1})]$$

These distributions correspond to uniform sampling over a logarithmic scale, ensuring equal weight across orders of magnitude. This reflects weakly informative prior assumptions: $RR_{CE}$ is at least equal in prevalence among vaccinated individuals and may be up to 10 times more common, while $RR_{CD}$ ranges from negligible association (RR = 1) to highly protective (RR = 0.01).

Using these priors, we computed the posterior distribution of the product $RR_{CE}\cdot RR_{CD}$ and estimated the probability that this product falls below a given $RR_{\text{obs}}$. In doing so, we obtained the probability that a confounder drawn from these prior assumptions could plausibly explain the observed data.

The analysis was implemented in MATLAB via Monte Carlo simulation. We defined latent parameters $RR_{CE}$ and RR_CD drawn from user-specified prior distributions, and computed the product $RR_{CE}\;\times \;RR_{CD}$ together with an indicator for whether the inequality $RR_{CE}\;\times \;RR_{CD}\;\leq \;RR_{obs}$ was satisfied. A total of 30000 samples were generated across three independent random seeds to assess the proportion of confounder configurations capable of fully explaining the observed effect. Since no likelihood function was specified, no posterior inference or convergence diagnostics were required. This simulation framework allows us to compute the proportion of draws where the Cornfield inequality is satisfied, quantifying how often unmeasured confounding of sufficient strength would arise under the specified assumptions.

## 3. Results

### 3.1. Cornfield inequality: Deterministic constraints on confounding

We first applied the Cornfield inequality to quantify the minimum strength of association between an unmeasured confounder and disease outcome (denoted $RR_{CD}$) that would be required to explain observed vaccine effectiveness estimates, assuming a range of plausible associations between the confounder and vaccination status ($RR_{CE}$). For instance, given an observed risk ratio of $RR_{\text{obs}}=\text{0}.\text{0}\text{8}$ — a value consistent with several early observational studies of the Pfizer-BioNTech vaccine [5] — the required $RR_{CD}$ rises sharply as $RR_{CE}$ decreases.

For each assumed value of the risk ratio between the confounder and the exposure ($RR_{CE}$), the table reports the minimum required risk ratio between the confounder and the disease ($RR_{CD}$) that would be necessary to fully account for the observed vaccine effect, according to the Cornfield inequality. As $RR_{CE}$ approaches 1 — that is, as the imbalance in confounder prevalence decreases — the required $RR_{CD}$ becomes increasingly extreme.

Table 1 shows the computed values for $RR_{CD}=RR_{\text{obs}}/RR_{CE}$, across values of $RR_{CE}$ ranging from 1.1 to 10. When $RR_{CE}=\text{2}$, the confounder would need to reduce disease risk by at least 96% ($RR_{CD}=\text{0}.\text{0}\text{4}$) to account for the observed association. If $RR_{CE}=\text{1}.\text{2}$ — a more realistic value for many demographic or behavioral confounders — then the required $RR_{CD}$ would be as low as 0.067, implying an unmeasured factor with stronger protective effect than any known intervention.

**Table 1:** Minimum strength of confounding required to explain an observed risk ratio of 0.08.

$RR_{CE}$	Required $RR_{CD}$
1.1	0.073
1.2	0.067
1.5	0.053
2.0	0.04
3.0	0.027
5.0	0.016
10.0	0.008

These calculations reveal a key insight: the more plausible the imbalance in confounder prevalence (i.e., $RR_{CE}$ is near 1), the more implausible the necessary effect size on disease risk becomes. In other words, any single confounder strong enough to fully account for the observed risk ratios must be both highly imbalanced and extremely protective, which is epidemiologically unlikely.

To partially address the uncertainty around observed VE estimates, we repeated the analysis using more conservative risk ratios (e.g., RR = 0.15) and found that even under weaker effects the required confounding strength remains implausibly high.

### 3.2. Sensitivity analysis: Proportion of confounders explaining the effect

While the Cornfield inequality provides a minimal threshold, it does not quantify how frequently such a confounder would arise under plausible assumptions. To assess this, we performed a Monte Carlo sensitivity analysis in which confounder strengths were sampled from broad, weakly informative distributions for $RR_{CE}$ and $RR_{CD}$. This allowed us to estimate the proportion of simulated confounder profiles capable of fully explaining the observed vaccine effect.

**Fig 1:**
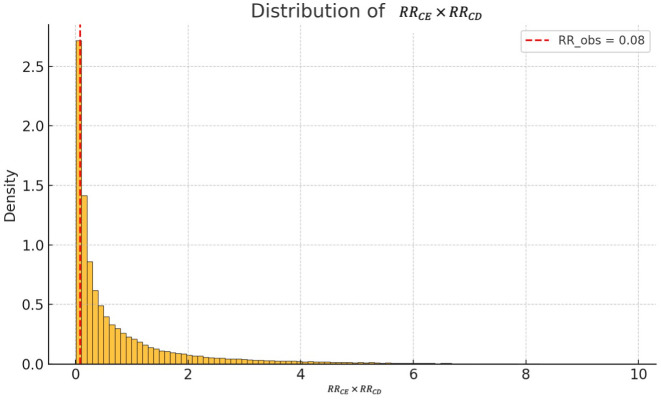
Distribution of the product $RR_{CE}\;\times \;RR_{CD}\;$ under log-uniform prior distributions for both parameters. The vertical dashed red line indicates the observed risk ratio ($RR_{obs}\;=\;\text{0}.\text{0}\text{8}$), associated with the Pfizer-BioNTech vaccine in several real-world studies. Only a small fraction of the simulated values lies to the left of this threshold, indicating that — under the specified prior assumptions — unmeasured confounding is unlikely to fully explain the observed effect (≈1.4% of simulations).

Fig 1 displays the distribution of the product $RR_{CE}\cdot RR_{CD}$, obtained via Monte Carlo sampling. The majority of the mass lies above 0.2, far above the threshold of $RR_{\text{obs}}=\text{0}.\text{0}\text{8}$. Only 1.4% of the simulated confounder configurations satisfied the condition $RR_{CE}\;\times \;RR_{CD}\;\leq \;\text{0}.\text{0}\text{8}$ was only 0.014 — meaning that under the chosen priors, only 1.4% of simulated confounders were capable of fully explaining the observed effect.

**Fig 2:**
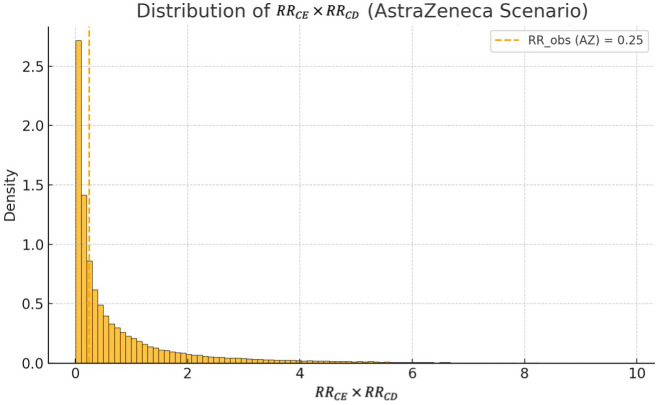
Distribution of the product $RR_{CE}\times RR_{CD}$ under the same prior assumptions, shown with reference to an observed risk ratio of $RR_{obs}=0.25$, corresponding to estimates reported for the AstraZeneca vaccine. Although the threshold is less extreme than in the Pfizer scenario, the majority of the distribution mass remains above it. The proportion of simulated confounder configurations capable of fully explaining this effect is approximately 5.8%, indicating that even in this more moderate case, a strong and implausibly structured confounder would be required.

This result holds consistently across different vaccine effectiveness levels. For instance, using an observed risk ratio of 0.25 — as reported for the AstraZeneca vaccine in several large-scale observational studies — the probability that an unmeasured confounder could fully explain the effect increases modestly to 5.8%. However, as shown in Fig 2, the bulk of the mass still lies above this threshold, reinforcing the conclusion that even more moderate risk reductions cannot be easily attributed to a hidden bias unless one assumes a confounder of implausible strength and imbalance.

These findings indicate that even under generous assumptions, it is statistically unlikely that an unmeasured confounder could solely account for the observed magnitude of vaccine protection. This simulation-based approach allows for a probabilistic statement: not only must a confounder be strong and imbalanced, but it must be implausibly so, given the assumptions specified in the prior distributions.

While this approach does not constitute a full Bayesian model with likelihood-based inference, it provides an informative summary of how often confounding of the required strength arises under plausible prior scenarios.

### 3.3. Sensitivity to prior assumptions

To test the robustness of our findings, we repeated the analysis using more permissive priors: specifically, allowing $RR_{CE}$ up to 20 and $RR_{CD}$ down to 0.001. Under this more extreme scenario, the probability that a confounder could explain an observed $RR_{\text{obs}}=\text{0}.\text{0}\text{8}$ increased modestly to 3.9%. However, the required values of $RR_{CD}$ and $RR_{CE}$ in these cases were so extreme — often implying a confounder that is 10 times more common among the vaccinated and reduces disease risk by 99% — that their plausibility remains questionable.

These findings reinforce the conclusion that the explanatory power of unmeasured confounding, while theoretically possible, is practically implausible in this context — especially when judged against known epidemiological patterns and biological mechanisms.

## 4. Discussion

Our analysis provides a quantitative response to a persistent criticism in observational epidemiology: the apprehension that unmeasured confounding may fully account for the observed vaccine efficacy. Although this is a plausible theoretical possibility, our findings indicate that such an explanation necessitates exceptionally robust and highly structured confounding—conditions that are both statistically improbable and biologically improbable.

Employing the Cornfield inequality, we demonstrated that for confounding alone to account for an observed risk ratio as low as 0.08, the confounder would necessitate either a significantly higher prevalence in the vaccinated population or an exceptionally robust protective effect against infection. For instance, if the disparity in confounder prevalence is moderate—approximately a 1.5-fold increase in the vaccinated group—the requisite risk ratio associated with the disease would have to be below 0.06. This would imply a level of protection surpassing any behavioral, demographic, or clinical factor currently recognized by scientific knowledge.

The sensitivity analysis further corroborated this conclusion. Instead of assuming confounding strength as a fixed unknown, we assigned priors that reflect our actual uncertainty regarding the plausible distribution of confounding relationships. This approach enabled us to estimate the probability that a confounder satisfying the Cornfield condition exists. In the case of the Pfizer-BioNTech vaccine, the probability that any confounder drawn from our prior could fully account for the observed effect was less than 2%. Even for more moderate vaccine effectiveness (e.g., AstraZeneca), this probability remained below 6%. These values are not zero, but they are sufficiently low to shift the burden of proof onto those asserting that confounding is the primary explanation.

Our findings do not assert that all observational estimates are immune to bias. Nor do they rule out the possibility that unmeasured variables may either diminish or enhance the effects of vaccines. Our argument is more nuanced: that the magnitude of the observed effects is too substantial to be plausibly attributed solely to unmeasured confounding, unless one accepts exceptionally stringent and specific assumptions. In this manner, our approach aligns with the logic of falsifiability in scientific reasoning: we are not substantiating a causal claim, but rather demonstrating that alternative non-causal explanations encounter a high evidential hurdle.

From an epistemological standpoint, our research integrates different methodologies. The Cornfield inequality serves as a prerequisite for causal inference, establishing a boundary that any non-causal explanation must surpass. Subsequently, the sensitivity framework quantifies the likelihood of this condition’s fulfillment under plausible beliefs. This amalgamation of deterministic and probabilistic reasoning facilitates a more transparent discourse on uncertainty and inference, particularly in contentious public health arenas where assertions frequently become politicized.

The E-value approach [9] offers an alternative framework for evaluating the impact of unmeasured confounding. Like the Cornfield inequality, it provides a threshold for the minimum strength of confounding needed to explain an observed effect. Our approach complements this by integrating a probabilistic quantification via simulation, which allows researchers to specify prior beliefs and assess the likelihood that such thresholds are actually exceeded.

Our approach also differs from the Monte Carlo bias analysis proposed by Greenland et al. [10], which simulates distributions of adjusted effect estimates under assumptions about the strength and prevalence of unmeasured confounders. Instead of generating corrected estimates, we focus on quantifying how frequently a confounding configuration would be strong enough to fully explain the observed effect. This distinction reflects a conceptual shift: rather than correcting estimates for potential bias, we assess the plausibility of complete confounding as an alternative explanation. In doing so, our method addresses a narrower but highly relevant question — namely, whether the observed association is “too large to be explained away,” even under generous assumptions.

Our analysis has certain limitations. We concentrated on a single binary confounder model, whereas real-world confounding may encompass multiple correlated factors. However, the inclusion of multiple confounders typically enhances, rather than diminishes, the stringency of the Cornfield condition [10]. Furthermore, our priors, although broad, remain subjective and could be subject to criticism as overly optimistic or pessimistic. Nevertheless, sensitivity analyses employing more permissive priors still yielded low probabilities of complete confounding.

In conclusion, we underscore that this methodology does not supplant randomized trials or meticulous study design. Instead, it complements them by offering a rational framework to discern instances where observational associations are “too substantial to be confounded.” In the context of COVID-19 vaccine efficacy, our findings indicate that the data are robust, not in the sense of being impervious to bias, but rather in the more practical sense that alternative explanations necessitate progressively implausible assumptions as effect sizes increase.

## 5. Conclusions

Unmeasured confounding remains a persistent concern in observational studies, especially when the findings have significant policy implications, such as those related to the effectiveness of COVID-19 vaccines. However, not all concerns are equally plausible. Our analysis, which integrates the classical Cornfield inequality with a sensitivity framework, demonstrates that attributing observed vaccine effects solely to confounding necessitates assumptions that approach the implausible.

In scenarios where vaccines demonstrate substantial protective effects (e.g., risk ratios approaching 0.1), any confounder capable of fully accounting for these outcomes must be both significantly more prevalent among the vaccinated and possess an unrealistically robust protective effect against infection. The simulation further elucidates that such a confounder is not only hypothetically sufficient but also statistically improbable, considering reasonable prior assumptions regarding population demographics.

These findings do not imply that observational data is equivalent to randomized evidence or that confounding is absent. Instead, they provide a quantitative argument for the robustness of the effect sizes reported for several COVID-19 vaccines and a framework for evaluating alternative explanations based on transparency and probabilistic reasoning.

In a broader perspective, our approach demonstrates how tools from both frequentist and simulation-based frameworks can synergistically support rational inference in high-stakes empirical contexts. When data indicate substantial effects and the competing hypotheses propose implausible mechanisms, the most plausible conclusion is that the effect is genuine, albeit with imperfect measurement.
